# Bacteroides vulgatus Ameliorates Lipid Metabolic Disorders and Modulates Gut Microbial Composition in Hyperlipidemic Rats

**DOI:** 10.1128/spectrum.02517-22

**Published:** 2023-01-10

**Authors:** Mingchao Xu, Ruiting Lan, Lei Qiao, Xiaoying Lin, Dalong Hu, Suping Zhang, Jing Yang, Juan Zhou, Zhihong Ren, Xianping Li, Guoxing Liu, Liyun Liu, Jianguo Xu

**Affiliations:** a Department of Epidemiology, Center for Global Health, School of Public Health, Nanjing Medical University, Nanjing, Jiangsu Province, China; b State Key Laboratory of Infectious Disease Prevention and Control, National Institute for Communicable Disease Control and Prevention, Chinese Center for Disease Control and Prevention, Beijing, China; c School of Biotechnology and Biomolecular Sciences, University of New South Wales, Sydney, New South Wales, Australia; d Research Units of Discovery of Unknown Bacteria and Function, Chinese Academy of Medical Sciences, Beijing, China; e Institute of Public Health, Nankai University, Tianjin, China; Shandong University

**Keywords:** *Bacteroides vulgatus*, hyperlipidemia, gut microbiota, bile acids, short-chain fatty acids

## Abstract

Hyperlipidemia is a risk factor and key indicator for cardiovascular diseases, and the gut microbiota is highly associated with hyperlipidemia. Bacteroides vulgatus is a prevalent mutualist across human populations and confers multiple health benefits such as immunoregulation, antiobesity, and coronary artery disease intervention. However, its role in antihyperlipidemia has not been systematically characterized. This study sought to identify the effect of B. vulgatus Bv46 on hyperlipidemia. Hyperlipidemic rats were modeled by feeding them a high-fat diet for 6 weeks. The effect of B. vulgatus Bv46 supplementation was evaluated by measuring anthropometric parameters, lipid and inflammation markers, and the liver pathology. Multi-omics was used to explore the underlying mechanisms. The ability of B. vulgatus Bv46 to produce bile salt hydrolase was confirmed by gene annotation and *in vitro* experiments. Oral administration of B. vulgatus Bv46 in hyperlipidemic rats significantly reduced the body weight gain, food efficiency, and liver index, improved the serum lipid profile, lowered the levels of serum inflammatory cytokines, promoted the loss of fecal bile acids (BAs), and extended the fecal pool of short-chain fatty acids (SCFAs), especially propionate and butyrate. B. vulgatus Bv46 induced compositional shifts of the gut microbial community of hyperlipidemic rats, characterized by a lower ratio of *Firmicutes* to *Bacteroidetes* with an increase of genera *Bacteroides* and *Parabacteroides*. After intervention, serum metabolite profiling exhibited an adaptation in amino acids and glycerophospholipid metabolism. Transcriptomics further detected altered biological processes, including primary bile acid biosynthesis and fatty acid metabolic process. Taken together, the findings suggest that B. vulgatus Bv46 could be a promising candidate for interventions against hyperlipidemia.

**IMPORTANCE** As a core microbe of the human gut ecosystem, Bacteroides vulgatus has been linked to multiple aspects of metabolic disorders in a collection of associative studies, which, while indicative, warrants more direct experimental evidence to verify. In this study, we experimentally demonstrated that oral administration of B. vulgatus Bv46 ameliorated the serum lipid profile and systemic inflammation of high-fat diet-induced hyperlipidemic rats in a microbiome-regulated manner, which appears to be associated with changes of bile acid metabolism, short-chain fatty acid biosynthesis, and serum metabolomic profile. This finding supports the causal contribution of B. vulgatus in host metabolism and helps to form the basis of novel therapies for the treatment of hyperlipidemia.

## INTRODUCTION

Hyperlipidemia, a pathological condition of lipid metabolism, is mainly manifested as elevated levels of total blood cholesterol (TC), triglyceride (TG), and low-density lipoprotein cholesterol (LDL-C) and a reduced level of high-density lipoprotein cholesterol (HDL-C) (1). The occurrence and development of hyperlipidemia are known as a contributing factor in metabolic syndromes, such as atherosclerosis and coronary heart disease as well as nonalcoholic fatty liver disease (2). However, its etiology is complex and the underlying mechanisms are not yet determined. The gut microbiota has been shown to be associated with hyperlipidemia for its regulatory roles in the storage, decomposition, and distribution of lipids (3). The microbiota-driven metabolites, such as short-chain fatty acids (SCFAs), lipopolysaccharide, bile acids (BAs), and trimethylamine N-oxide, among others, may serve as key signaling molecules that couple the gut flora with the host (4–6). Therefore, targeting gut microbiota may be an effective strategy in the treatment of hyperlipidemia.

Data from animal models and human volunteers with hyperlipidemia or related dysfunctions showed that the relative abundance of specific microbial communities, such as *Bacteroides*, *Parabacteroides*, and *Faecalibacterium* (7–10), was inversely proportional to disease status. These findings provided the basis for a more targeted therapeutic strategy that focuses on gut microbiota in hyperlipidemia intervention. As one of the global human core microbiota members (11), *Bacteroides*, featuring an unusual genetic endowment (polysaccharide utilization loci) to metabolize dozens of plant- and host-derived polysaccharides (12), is generally considered a promising commensal for its powerful adaptive characteristics and underlying benefits in governing intestinal ecological function and stability (13, 14).

As a prominent species of the genus *Bacteroides*, Bacteroides vulgatus has been described as a prevalent and dominant organism of the colon in our previous work (15) and other studies (16, 17). B. vulgatus is equipped with enzyme systems to degrade complex polysaccharides (18) and is known to produce acetate, propionate, butyrate, and lactate (19, 20). To date, B. vulgatus has been intensively explored as a treatment for intestinal inflammatory diseases, while its effects are strain dependent (21, 22). Recently, a few studies have shown the potential benefits of B. vulgatus in metabolic disorders: for example, 16S rRNA gene analysis of fecal samples from patients with coronary artery disease showed a significant decrease in the relative abundance of B. vulgatus and Bacteroides dorei, and oral administration of B. vulgatus and *B. dorei* could suppress microbial lipopolysaccharide production and attenuate atherosclerotic lesion formation in mice (23). With respect to obesity, the combination of B. vulgatus and *B. dorei* enhanced the catabolism of branched-chain amino acids in brown adipose tissue and reduced the body weight gain in mice with diet-induced obesity (24).

Given the evidence that B. vulgatus plays a role in lipid metabolism and exerts possible beneficial effects in reducing obesity, in this study, we employed a combination of microbiome analyses, metabolomics, and transcriptomics to investigate the protective effect of B. vulgatus against hyperlipidemia in the rat model in a high-fat diet setting.

## RESULTS

### BSH activity.

Upon genome annotation, the draft genome of B. vulgatus Bv46 was found to encode a choloylglycine hydrolase family protein (MCG0330840.1), which was also designated bile salt hydrolase (BSH; EC 3.5.1.24), involved in the hydrolysis of conjugated bile salts (25). Consistently, in the standard plate assay, B. vulgatus Bv46 formed halos and white precipitates surrounding colonies (see Fig. S1 in the supplemental material), similarly to the positive reference strain Lactobacillus gasseri Y20 (26), indicating the production of the BSH enzyme.

### Effect of B. vulgatus Bv46 on anthropometric and biochemical parameters.

**FIG 1:**
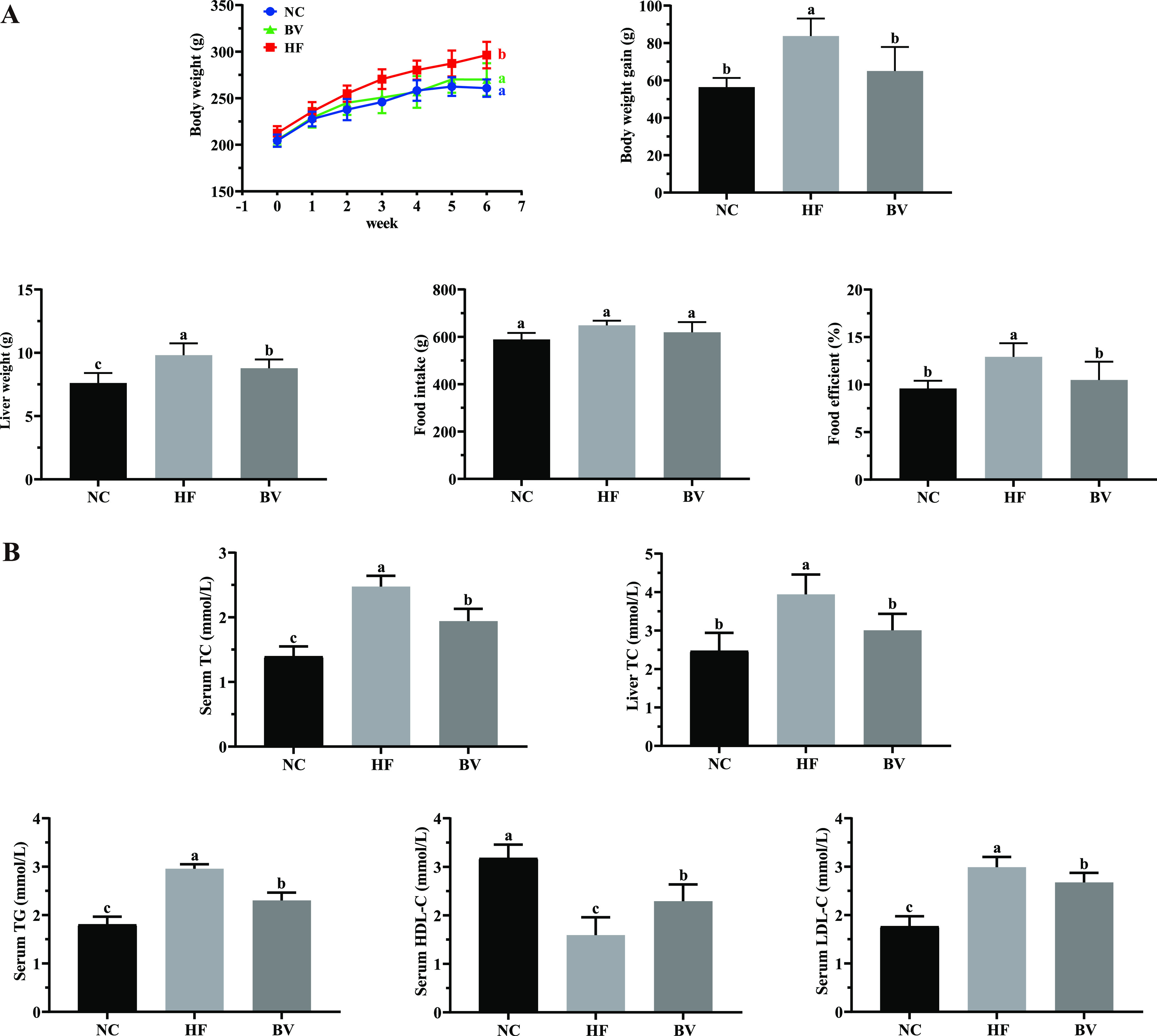
Effect of B. vulgatus Bv46 supplementation on anthropometric and biochemical parameters in rats fed with a high-fat diet. (A) Body weight change, body weight gain, liver weight, food intake, and food efficiency. (B) Serum TC, TG, HDL-C, and LDL-C and liver TC. Statistical comparison was performed by a one-way ANOVA followed by Tukey’s multiple-comparison test. Values are expressed as the mean ± standard error of the mean in each group; different letters (a to c) are considered significantly different at a *P* value of <0.05. NC, normal-chow diet group; HF, high-fat diet group; BV, group with a high-fat diet plus B. vulgatus Bv46.

**FIG 2:**
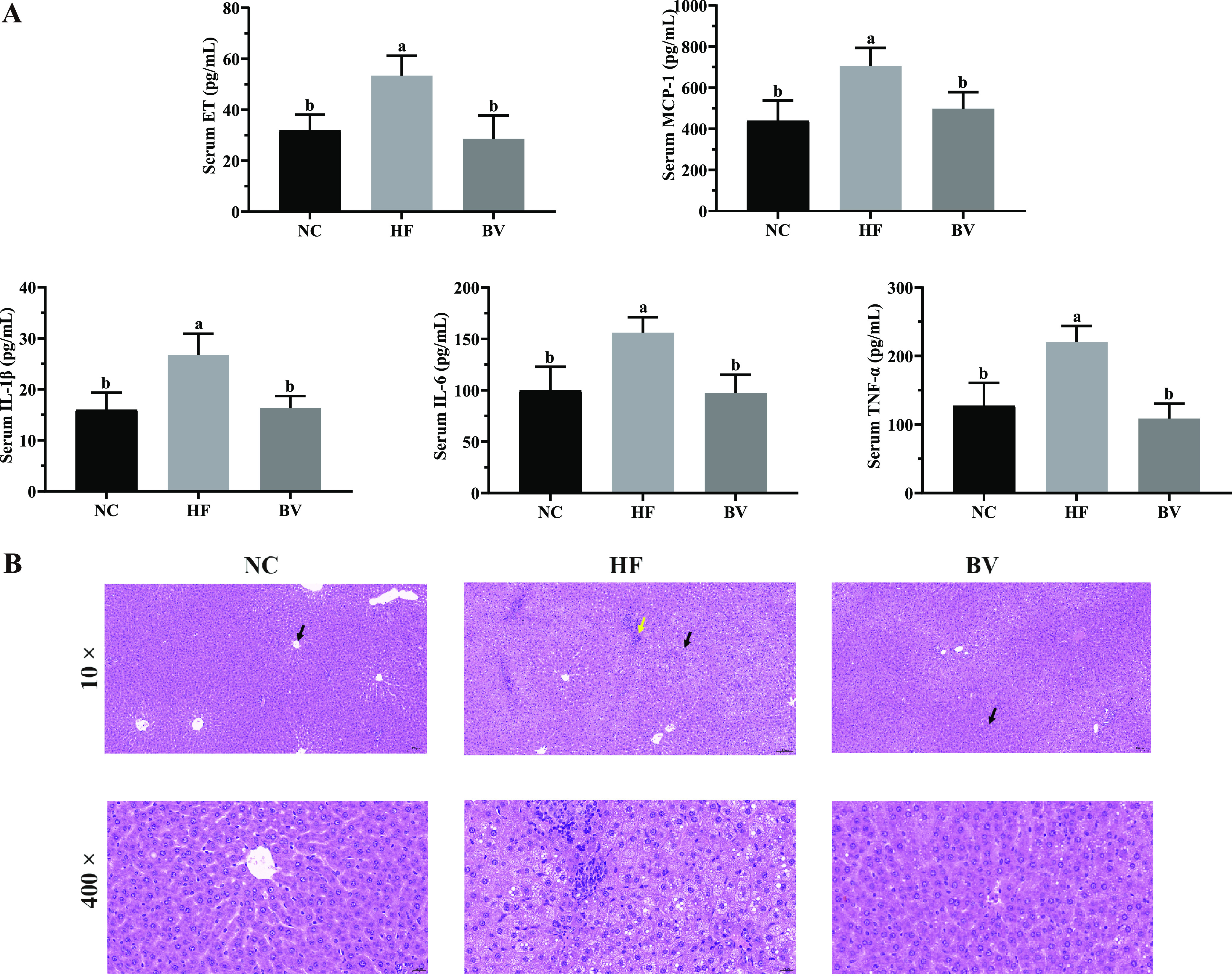
Effect of administration of B. vulgatus Bv46 on systemic inflammation and hepatic lipid deposition. (A) Levels of serum ET, MCP-1, IL-1β, IL-6, and TNF-α. (B) Representative images of liver tissues stained with H&E. Black arrows point to areas which were magnified 400 times and are shown in the bottom panel. The yellow arrow indicates the presence of inflammatory infiltration. Statistical comparison was performed by a one-way ANOVA followed by Tukey’s multiple-comparison test. Values are expressed as the mean ± standard error of the mean in each group; different letters (a to c) are considered significantly different at a *P* value of <0.05.

As expected, the body weight of the high-fat (HF) group rats was significantly higher than that of the normal control (NC) group rats (*P* < 0.05) from the 3rd week onward (Fig. 1A). After 6 weeks of modeling, the HF group exhibited a significant increase in body weight gain and liver weight compared to the NC and B. vulgatus (BV) groups (*P* < 0.05), and the body weight gains were comparable between the BV and NC groups (*P* > 0.05). Although the values for cumulative food intake per rat were almost similar among the three groups (*P* > 0.05), the food efficiency ratio of the BV group was lower than that of the HF group (*P* < 0.05; Fig. 1A). In addition, the semiquantitative hematoxylin-and-eosin (H&E) staining of liver sections in the HF group of rats showed extensive cytoplasmic vacuolation and less-organized structure as well as inflammatory cell infiltration. These adipopathological phenomena were diminished with the supplementation of B. vulgatus Bv46 (Fig. 2B).

Compared with the NC group, consumption of the high-fat diet led to a marked rise in serum TC, TG, and LDL-C concentrations (*P* < 0.05) and a significant reduction of serum HDL-C (*P* < 0.05). Notably, these derailed serum lipid profiles were averted in rats administered B. vulgatus Bv46. Similar to the level in serum, TC level in the liver, an indicator of hepatic lipid accumulation, substantially declined in the BV group (*P* < 0.05; Fig. 1B). Moreover, as shown in Fig. 2A, serum levels of interleukin 1β (IL-1β), interleukin 6 (IL-6), tumor necrosis factor alpha (TNF-α), monocyte chemotactic protein 1 (MCP-1), and endotoxin (ET) were increased in the HF group compared to the NC group (*P* < 0.05). After 6 weeks of B. vulgatus Bv46 intervention, the expression of the above-mentioned proinflammatory cytokines and the circulating level of ET were significantly inhibited (*P* < 0.05).

### Regulation by B. vulgatus Bv46 of gut microbiota dysbiosis.

**FIG 3:**
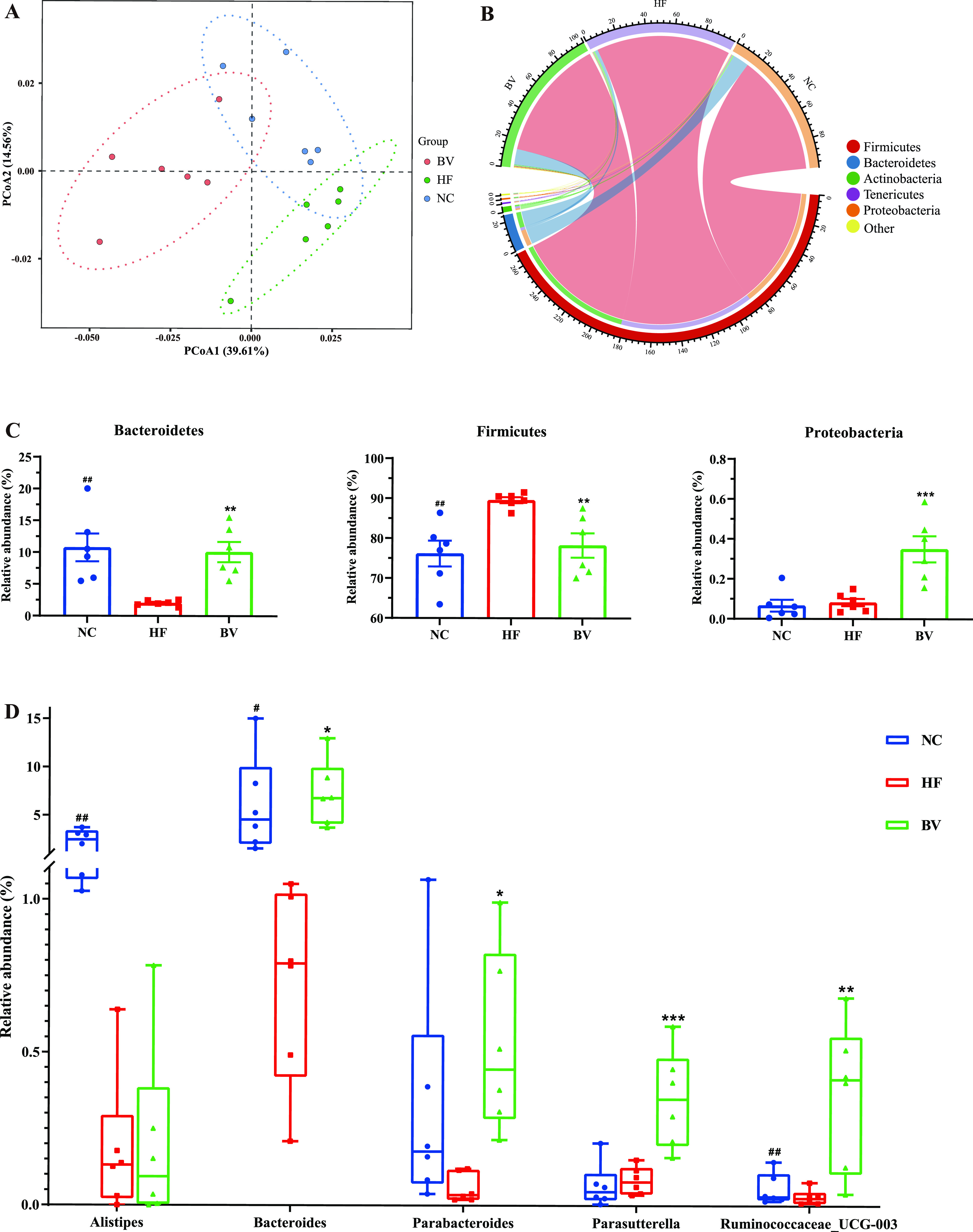
Fecal microbiota alterations of different groups. (A) Principal-coordinate analysis (PCoA) of β-diversity pattern based on weighted UniFrac distance. (B) Composition of predominant bacterial communities at phylum level. (C) Comparison of the relative abundance of major phyla between groups. ##, *P* < 0.01, indicates the significant differences of the HF group from the NC group, and **, *P* < 0.01, and ***, *P* < 0.001, indicate significant differences from the BV group. (D) Relative abundance of bacterial genus differentially enriched in each group. The differences between groups were compared using *t* test or Welch’s *t* test, and the Storey *q* value was employed to control the false-discovery rate due to multiple testing. Data are exhibited as quartiles. #, *q* < 0.05, and ##, *q* < 0.01, indicate the significant differences of the HF group from the NC group, and *, *q* < 0.05, **, *q* < 0.01, and ***, *q* < 0.001 indicate differences from the BV group.

To investigate diet-induced shifts in gut bacterial communities, high-throughput 16S rRNA amplicon sequencing was conducted using rats’ fecal samples. Rarefaction analysis showed that the unigene sets reached the asymptotical plateau (Fig. S2A), suggesting that all samples had good microbial community coverage. As for intrasample diversity (α-diversity), the bacterial richness (Chao1) was lower in the HF and BV groups than in the NC group (*P* < 0.05), while no significant differences were found among the three groups in the Pielou evenness index and Shannon index (*P* > 0.05), which took both bacterial richness and evenness into consideration (Fig. S2B). As illustrated by the principal-coordinate analysis based on the weighted UniFrac algorithm (Fig. 3A), the β-diversity characterizing the intersample relationship of the microbial compositions was affected by diets: rats fed with two high-fat diets were separately clustered from the NC group, and B. vulgatus Bv46-treated rats were clearly segregated from those in the HF group. Permutational multivariate analysis of variance (PERMANOVA) multidimensional statistical analysis further confirmed the differences among the three groups (NC versus HF, *R *= 0.196, *P* = 0.004; NC versus BV, *R *= 0.309, *P* = 0.002; HF versus BV, *R *= 0.248, *P* = 0.045), which indicated that the intake pattern of B. vulgatus Bv46 affected the overall gut microbial structure of high-fat diet-fed rats.

It was observed that the vast majority of fecal microbiota in all groups were made up of *Firmicutes* and *Bacteroidetes* (Fig. 3B). Compared with the NC group, the abundance of *Firmicutes* was higher, while the *Bacteroidetes* was lower, in the HF group (*P* < 0.05; Fig. 3C). Significantly, treating rats with B. vulgatus Bv46 reduced the ratio of *Firmicutes* to *Bacteroidetes* (F/B ratio; *P* < 0.05) in high-fat diet-feeding rats (Fig. S2C) and resulted in a higher relative abundance of the phylum *Proteobacteria* (*P* < 0.05; Fig. 3C). At genus level, the relative abundance of zero-distance operational taxonomic units (zOTUs) related to *Alistipes*, *Bacteroides*, and *Ruminococcaceae_UCG-003* was significantly higher in the NC group than in the HF group (*q *< 0.05; Fig. 3D). Biologically consistent differences were presented between the two high-fat diet consumption groups. After 6 weeks of B. vulgatus Bv46 intake, the relative abundance of the genera *Bacteroides* and *Parabacteroides* was dramatically increased (*q *< 0.05), which substantially contributed to the differential enrichment of the *Bacteroidetes*. Besides, it also induced an increase of the genera *Parasutterella* and *Ruminococcaceae_UCG-003* (*q *< 0.05; Fig. 3D). As for the prevalence pattern of B. vulgatus in response to gavage, we performed B. vulgatus species-specific quantitative PCR (qPCR) and confirmed that the abundance of B. vulgatus in feces of B. vulgatus Bv46-fed rats was higher than that of the HF group (*P* < 0.05; Fig. S2D).

Moreover, PICRUSt2 was used to predict the functional changes of the intestinal microbiota induced by B. vulgatus Bv46. Analysis of the abundance distribution of pathways showed that metabolic functions like beta-alanine metabolism, taurine and hypotaurine metabolism, and phosphonate and phosphinate metabolism were upregulated in the BV group (*q *< 0.05), while insulin resistance was upregulated in the HF group (*q *< 0.05; Fig. S2E).

### Endogenous metabolites altered by B. vulgatus Bv46.

**FIG 4:**
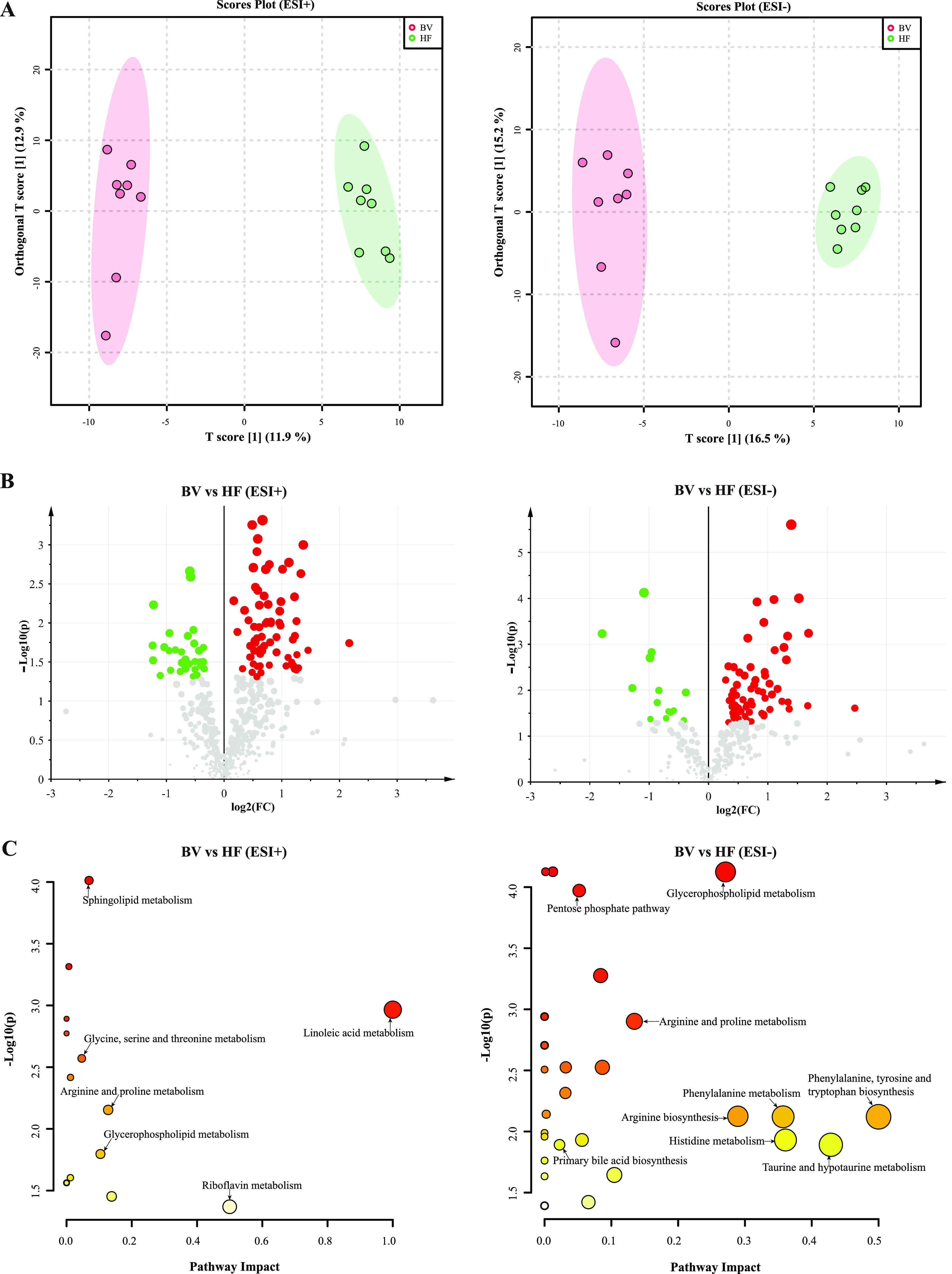
Serum metabonome changes of hyperlipemia rats in the HF and BV groups (ESI^+^, left column; ESI^−^, right column). (A) Orthogonal partial least-squares discriminant analysis (OPLS-DA) plots. (B) The volcano plots of differential metabolites (ESI^+^, 99; ESI^−^, 84) in the serum; the red dots represent an upregulation of the relative abundance of serum metabolites, the blue ones represent downregulation, and gray dots represent no significance. The size of dots represents the variable importance plot value (VIP). (C) The scatterplots of 44 enriched KEGG pathways (*q *< 0.05) based on significantly altered metabolites. The color of solid circles indicates the different level of significance (the ordinate) in enrichment analysis. The size of the circles represents the impact score (the abscissa) of the corresponding pathway.

Considering the distinct physiological indices conveyed by dietetic intervention, the metabolic status of serum was examined using ultrahigh-performance liquid chromatography–tandem mass spectrometry (UHPLC-MS/MS). A separate clustering pattern was illustrated for the three groups in partial least-squares discriminant analysis (PLS-DA) score plots (Fig. S3A and B). To explore the causative factors contributing to the distinction of the HF and BV groups, orthogonal partial least-squares discriminant analysis (OPLS-DA) models were built (Fig. 4A). As shown in Fig. 4B, 183 metabolites were identified to be significantly changed (variable importance plot [VIP] values > 1.0 and *P* < 0.05) in the electrospray ionization-positive (ESI^+^; basic species) and -negative (ESI^−^; acidic species) modes. Among them, compared with those in rats in the HF group, levels of 138 metabolites were increased in the BV group. Through pathway enrichment, part of the differential features was matched to KEGG metabolic pathways. The high-fat diet induced comprehensive changes in amino acids, lipids, and carbohydrate metabolism such as taurine and hypotaurine metabolism, primary bile acid biosynthesis, and tyrosine, tryptophan, and histidine metabolism as well as glycerophospholipid metabolism (Fig. S4 and Tables S4 and S5), compared with the normal-chow diet group. As expected, hyperlipidemia rats under B. vulgatus Bv46 treatment showed repaired metabolomic features, including elevations in serum taurine, l-phenylalanine, l-histidine, carnosine, beta-alanyl-N(pi)-methyl-l-histidine, betaine, and creatine and reductions in serum phosphatidylcholine, 1-acyl-*sn*-glycero-3-phosphocholine, and phosphatidate (VIP values > 1.0 and *P* < 0.05; Fig. 4C and Tables S2 and S3).

**FIG 5:**
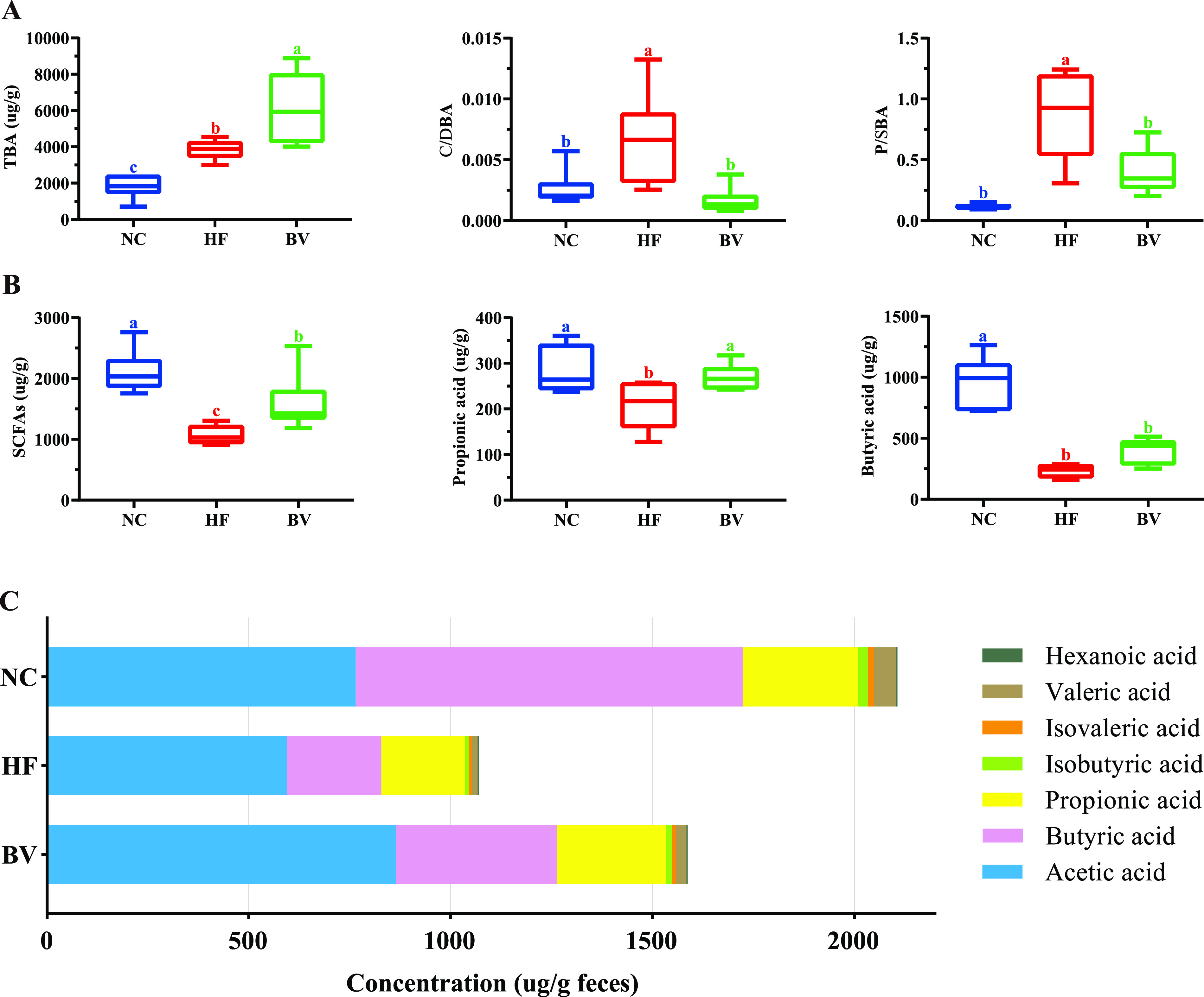
Analysis of feces BAs and SCFAs. (A) Fecal level of total BAs (TBA) and ratios of conjugated BAs to deconjugated BAs (C/DBA) and primary BAs to secondary BAs (P/SBA). (B) Fecal levels of SCFAs, propionic acid, and butyric acid. (C) Comparison of SCFA pool sizes and compositions among the three groups. The different SCFAs are represented by colors as shown in the color key. Statistical comparison was performed by testing normality first with the Kolmogorov-Smirnov test and then one-way ANOVA with Tukey’s multiple-comparison test or the Kruskal-Wallis test with Dunn’s *post hoc* test. Values are expressed as quartiles in each group; different letters (a to c) are considered significantly different at a *P* value of <0.05.

To quantify the direct impact of intestinal flora on specific metabolic features, further target profiling of BAs and SCFAs in feces was performed. Based on the obtained data set covering 30 types of BAs, different diets had a significant remodeling effect on the composition of the BA pool, as described by multivariate statistical analyses (Fig. S3C). Generally, the levels of 12 types of individual BAs were significantly increased in rats of the HF group compared with those in the NC group (VIP values > 1.0 and *P* < 0.05), and B. vulgatus Bv46 feeding resulted in elevated levels of 2 types of individual BAs compared with the HF group (VIP values > 1.0 and *P* < 0.05; Table S6). For the concentration of total BAs, there was a significant increase in the BV group over that in the HF group (*P* < 0.05). In terms of biotransformation, the high-fat diet increased the ratio of primary to secondary BAs and conjugated to unconjugated BAs compared to the normal-chow diet (*P* < 0.05), while this trend was significantly reversed by B. vulgatus Bv46 supplementation (*P* < 0.05; Fig. 5A). Likewise, the contents of total SCFAs (acetic acid, propionic acid, isobutyric acid, butyric acid, isovaleric acid, valeric acid, and hexanoic acid) excreted by rats in the BV group were higher than those by the HF group (*P* < 0.05). Notably, this study revealed an upregulation of propionic acid (*P* < 0.05) and a strong tendency toward increased butyric acid (*P* = 0.053) in the BV group rats compared with the HF group (Fig. 5B and C).

### Hepatic transcriptome changes in response to B. vulgatus Bv46.

**FIG 6:**
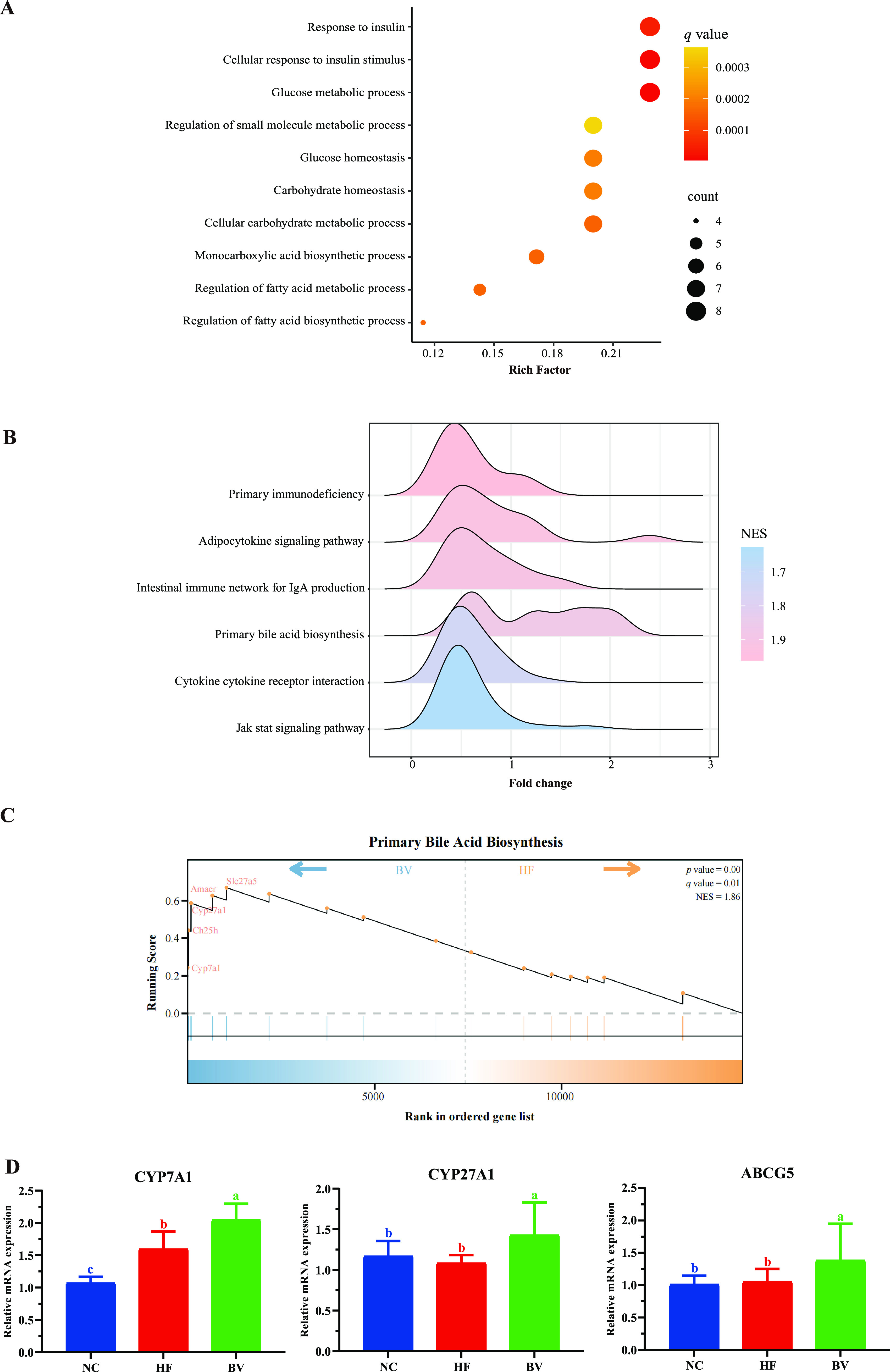
Hepatic gene expression alterations of rats with hyperlipidemia under B. vulgatus Bv46 treatment. (A) Gene ontology (biological process) enrichment analysis of the differentially expressed genes (DEGs) between the BV and HF groups. The count refers to the number of DEGs belonging to the corresponding pathway. The enrichment factor (Rich factor) is the ratio of DEGs in this pathway term to all annotated genes in the pathway term. (B) Overview of B. vulgatus Bv46-induced changes of metabolic and immune-related KEGG pathways (|NES| > 1, *q* < 0.05) identified by gene set enrichment analysis (GSEA); the distribution of core enriched genes for GSEA enriched pathways is visualized by the ridgeline plots and plotted by fold changes on the horizontal axis. (C) The position of core enriched genes of the primary bile acid biosynthesis pathway in the prior sorting list per comparison. (D) Quantitative real-time PCR validation of relative mRNA expression of CYP7A1, CYP27A1, and ABCG5 genes. GAPDH was used as an endogenous reference gene. Statistical comparison was performed by a one-way ANOVA followed by Tukey’s multiple-comparison test. Values are expressed as the mean ± standard error of the mean in each group; different letters (a to c) are considered significantly different at a *P* value of <0.05.

RNA sequencing (RNA-Seq) of liver tissues was used to disclose the protective role of B. vulgatus Bv46 and the related downstream mechanisms. Differential expression analyses found that compared with the HF group, 46 differentially expressed genes (DEGs) were detected in the BV group, with 30 genes being upregulated and 16 downregulated (Table S7). Functional allocation using gene ontology (GO) terms indicated that DEGs were mainly involved in metabolic processes including the glucose metabolic process, regulation of the fatty acid metabolic process, and the cellular carbohydrate metabolic process (Fig. 6A and Table S8).

Gene set enrichment analysis (GSEA) of KEGG pathways found that gene lists involved in primary bile acid biosynthesis, adipocytokine signaling, and immune-related pathways were activated by B. vulgatus Bv46 (Fig. 6B and C and Table S9). To determine the transcriptional pattern of BA metabolism induced by B. vulgatus Bv46, two differently expressed genes (Cyp7a1 and ABCG5) and a core enriched gene (Cyp27a1) were validated by quantitative real-time PCR. Consistently, the expression of Cyp7a1, Cyp27a1, and ABCG5 at mRNA level was significantly upregulated in the BV group compared to that in the HF group (*P* < 0.05; Fig. 6D).

## DISCUSSION

Accumulating evidence indicated tight interactions between the gut microbiota and hyperlipidemia and its metabolic comorbidities (3). A series of metagenome-wide association studies have shown that the abundance of certain *Bacteroides* species was depleted in patients with hyperlipidemia-related metabolic phenotypes, suggesting the imperativeness to assess the mutualistic roles of *Bacteroides* in metabolic syndromes (8, 9, 27, 28). Uniformly, gavage with Bacteroides acidifaciens, *B. dorei*, B. vulgatus, Bacteroides thetaiotaomicron, and Bacteroides uniformis was shown to alleviate different metabolic disorders such as adiposity and atherosclerosis (29, 30). Among the *Bacteroides* species, B. vulgatus, mainly involved in immunomodulatory effects as found previously (31, 32), has been implicated as being a novel microbial therapeutic target for intervention in metabolic dysfunctions (23, 24). However, its potential as a protective factor for hyperlipidemia had not been systematically investigated so far. The current study presents evidence that B. vulgatus Bv46 was able to ameliorate diet-induced hyperlipidemia and associated dysfunctions in rats. A multi-omics strategy, integrating 16S rRNA amplicon sequencing and untargeted and targeted metabolomics and transcriptomics resolutions, provided clues regarding the key mechanisms of the beneficial roles that B. vulgatus Bv46 played in antihyperlipidemia.

16S rRNA analysis showed that administration of B. vulgatus Bv46 reshaped the gut microbiome unbalanced by a high-fat diet at the phylum level, with an increased *Bacteroidetes* and decreased *Firmicutes* level, along with the reduced F/B ratio, which is generally observed in healthy individuals without obesity or metabolic syndrome (33, 34). Besides, a higher proportion of the *Firmicutes* phylum has been reported to be associated with harvesting of more energy (35, 36). Thus, the microbiota pattern with a lower F/B ratio may contribute to the food efficiency-lowering and antiobesity effect of B. vulgatus Bv46. At genus level, as expected, intragastric perfusion of B. vulgatus Bv46 significantly enhanced the abundance of *Bacteroides*, a primary commensal inhabitant in the gastrointestinal tract that can resist up to 20% bile salt (13). Congruously, as a representative species of *Bacteroides* spp. (17), B. vulgatus had shown BSH activity (37) and deconjugated taurodeoxycholic acid sodium salt in this study, which may confer a survival advantage in the mammal intestine characterized by intense bile toxicity (38). BSH has been also reported in members of the genus *Parabacteroides* (25, 39), which was enriched in the BV group in this study.

It is well established that BSH initiates the gateway reaction for further biotransformation of BAs involved in the regulation of lipid absorption, cholesterol homeostasis, and immunological balance in the intestine (37, 40). More specifically, BSH catalyzes the deconjugation of BAs to liberate primary BAs and glycine/taurine moiety. Deconjugated BAs are less soluble than their conjugated counterparts and easily precipitate as well as coprecipitate with cholesterol rather than be reabsorbed into enterohepatic cycling under acidic conditions. Therefore, compensatory BAs are *de novo* synthesized from cholesterol (41, 42). Subsequently, part of primary BAs is modified by the gut microbiota by means of dehydroxylation, dehydrogenation, and sulfation (43), which generates the secondary BAs. In this research, the dense BSH activity of gut flora enriched with B. vulgatus Bv46 was likely to promote increased BA deconjugation, as a lower ratio of conjugated to unconjugated BAs was observed in the feces of the BV group of rats. As a consequence, a larger amount of BAs was eliminated with the feces or transformed to the secondary BAs.

The enrichment plot from the comparison of enriched KEGG gene sets between the BV and HF groups showed that the primary bile acid biosynthesis pathway was upregulated in the BV group, and the core enriched genes Cyp7a1 and Cyp27a1 were overrepresented as validated by qPCR. Cyp7a1 and Cyp27a1 encode cytochrome P450 enzymes that function as the initiator of the neutral and acidic pathway of bile acid biosynthesis (44); upregulation of these genes would enhance the biological transformation from cholesterol to BAs, resulting in reduced cholesterol levels. Also of note, B. vulgatus Bv46 clearly promoted the expression of ABCG5, a sterol efflux transporter belonging to the ATP-binding cassette transporter superfamily responsible for the excretion of liver cholesterol into the bile (45), which may also reduce the liver cholesterol level.

The metabolic interplay between the host and gut microbiota has been shown to play a role in the pathogenesis of metabolic disorders; as an intermediate phenotype, circulating metabolites may afford a functional readout of the intestinal microbiome (46, 47). In this investigation, differential analysis of serum metabolites reported a significantly increased taurine in hyperlipidemia rats gavaged with B. vulgatus Bv46. Taurine is referred to as a “very essential” amino acid, which is endogenously synthesized from sulfur-containing amino acids such as cysteine and methionine or absorbed from food in the intestine (48). Of note, taurine can be used as the substrate for the formation of conjugated BAs, and taurine feeding induces an increase of BA pool size and the rate of its excretion (49). Taurine is also involved in various physiological processes, including antioxidation, immunoregulation, calcium transport, and lipid homeostasis (50). Both human and animal studies have confirmed the negative correlation between the plasma taurine content and the prevalence of obesity and diabetes (51), and taurine supplementation elevates the plasma taurine concentration and blunts the progression of metabolic dysfunctions such as nonalcoholic fatty liver disease, hyperlipidemia, diabetes, and obesity (52, 53). Concomitantly, PICRUSt2 analysis based on 16S rRNA gene sequences predicted that the changes in intestinal bacteria induced by B. vulgatus Bv46 supplementation would correspond to upregulation of KEGG functional category taurine and hypotaurine metabolism; thus, it is likely that the high level of taurine driven by the reshaped gut microbiota contributed to the retarded state of hyperlipidemia in high-fat diet-feeding rats. However, mechanisms responsible for the alteration of serum taurine remain to be further investigated.

Enrichment analysis of differential metabolites highlighted the pathways involved in amino acid metabolism, such as phenylalanine, tyrosine, and tryptophan biosynthesis, histidine metabolism, and glycine, serine, and threonine metabolism. To date, the salutary role of aromatic amino acids (phenylalanine, tryptophan, and tyrosine) and microbial aromatic amino acid metabolites has been linked to different biological processes, including immune cell response, triglyceride metabolism, and neuronal responses (54). In this study, l-phenylalanine implicated in the biosynthesis and metabolism of tyrosine and phenylalanine was significantly upregulated in BV group rats, a finding which was concordant with the favorable promise of l-phenylalanine in animals suffering from hyperlipidemia or obesity (55–57). B. vulgatus Bv46 replenishment also altered histidine metabolism: in particular, it significantly elevated levels of serum l-histidine, carnosine, and beta-alanyl-N(pi)-methyl-l-histidine in rats with high-fat diet-induced hyperlipidemia. l-Histidine is a standard proteinogenic amino acid that mammals do not synthesize *de novo*, and low levels of histidine can be synthesized by most intestinal bacteria or produced from carnosine in the blood of humans (58). To satisfy the histidine requirement of hosts, a daily intake of 4 g of l-histidine is recommended (59). However, serum l-histidine is often decreased in obese subjects (60). Carnosine and its methylated metabolite beta-alanyl-N(pi)-methyl-l-histidine (anserine) have a unique role in reducing lipid oxidation and removing reactive oxygen species (61); it can be speculated that the alteration of histidine-related substances following B. vulgatus Bv46 intervention may be beneficial as they ameliorate metabolic dysfunction. Glycine, serine, and threonine metabolism is connected to glycerophospholipid metabolism via choline and betaine as intermediates (62). Choline can be converted to phosphatidylcholine or phosphatidate or irreversibly oxidated to betaine (63). Betaine is a methyl derivative of glycine that protects against both alcohol-induced and metabolism-associated liver diseases by activating fatty acid transport and oxidation and reducing triglyceride and cholesterol accumulation as well as preserving intestine integrity (64). Our finding of a higher level of betaine and a lower level of phosphatidylcholine in the BV group than in the HF group further confirmed the lipid-lowering effect of B. vulgatus Bv46.

SCFAs, the secondary metabolites of anaerobic intestinal microbiota by fermentation of nondigestible carbohydrates, are saturated aliphatic organic acids with acetate, propionate, and butyrate as the major components (≥95%) (65). Depending on the diet and health status, their total volume varies among individuals. In this work, the total SCFAs detected in stool were shown to be lower in the high-fat diet-fed rats than in rats fed the normal-chow diet. In line with previous reports (21, 66), B. vulgatus Bv46 addition boosted the production of fecal SCFAs compared to that for high-fat feeding alone; this may indicate a lower intracolonic pH, which favored the coprecipitation of cholesterol with the deconjugated bile salts and promoted the generation of the BSH enzyme by the bacteria (42) and consequently contributed to the elimination of cholesterol by the host. Further, the elevation of propionate in the feces was in accord with the observed changes in the genera *Bacteroides* and *Parabacteroides* in response to B. vulgatus Bv46 intake, as both genera are able to produce propionate (4, 67). In the literature, propionate has been demonstrated to trigger the secretion of glucagonlike peptide 1 (GLP-1) and leptin, which could increase satiety and energy expenditure and suppress body weight gain (68, 69). On the other hand, propionate is classically featured as a gluconeogenic substrate in the intestine and the liver, and the process of intestinal gluconeogenesis can also be induced by butyrate (70), an energy substrate for colonocytes and enterocytes. Higher levels of propionate and butyrate resulting from B. vulgatus Bv46 intervention may accelerate the intestinal gluconeogenesis, which would benefit energy homeostasis and decrease food intake (71). The health benefits of SCFAs are also correlated with immune homeostasis. A recent study has shown that propionate could attenuate dyslipidemia by increasing the regulatory T-cell number and interleukin 10 level of the intestinal microenvironment (72). Butyrate is known to serve as an anti-inflammatory mediator by suppressing nuclear factor kappa B (NF-κB) through inhibition of histone deacetylase activity *in vitro* (73), and in metabolic disturbance models, butyrate intervention reduced the production of proinflammatory cytokines IL-1β, IL-6, MCP-1, and TNF-α (74, 75). Besides, propionate and butyrate were also reported to normalize the serum endotoxin level in high-fat diet-fed mice (74, 76); endotoxin is a complex molecule that augments the inflammatory cell infiltrations and aggravates the systemic inflammatory responses induced by a high-fat diet (77, 78). In this study, levels of proinflammatory cytokines as well as endotoxin were significantly reduced in the BV group, which was consistent with previous results (23), suggesting that the immune regulation role of B. vulgatus Bv46 may be attributable to microbiota-derived metabolites and components, such as propionate, butyrate, and endotoxin.

**FIG 7:**
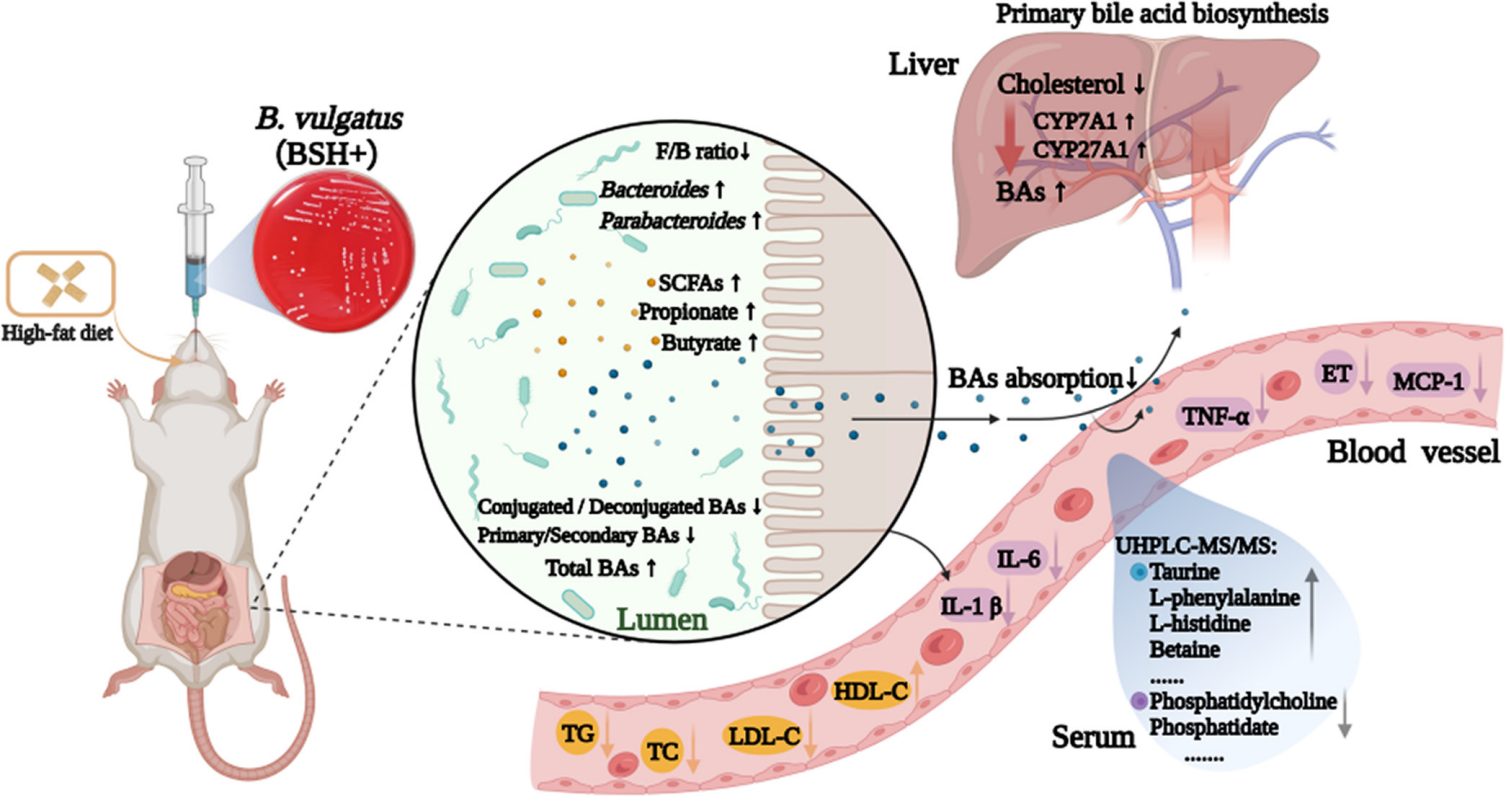
Schematic representation of antihyperlipidemia effect of B. vulgatus Bv46 on high-fat diet-fed rats.

Overall, the findings of this study provided strong evidence regarding the antihyperlipidemia effect of B. vulgatus Bv46. Mechanistically, treatment with B. vulgatus Bv46 restored the intestinal flora composition, modulated the metabolic profile, and enlarged the BA and SCFA pools, which accelerated energy expenditure, mitigated systemic inflammation, promoted cholesterol excretion, and improved lipid homeostasis of high-fat diet-feeding rats (Fig. 7). Collectively, this work provides avenues for further testing, either evaluating causative roles of differential metabolic biomarkers in hyperlipidemia development and functional roles of closely related bacteria such as *Parabacteroides* spp. or warranting the preventative strategies against metabolic dysfunction by expanding the abundance of *Bacteroides* spp.

## MATERIALS AND METHODS

### Bacterial cultures and bile salt hydrolase activity.

B. vulgatus Bv46 was recovered from healthy human feces collected previously (15) and deposited in the China General Microbiological Culture Collection Center (CGMCC) with preservation number CGMCC no. 17140. For activation, the strain was anaerobically subcultured at 37°C for 48 h on brain heart infusion (BHI) agar supplemented with 5% defibrinated sheep blood. Genomic DNA of B. vulgatus Bv46 extracted using the Wizard genomic DNA purification kit (Promega) was sequenced on the Illumina HiSeq TM2000 platform and assembled as outlined previously (79). The Illumina-based draft genome sequence of B. vulgatus Bv46 was submitted to the National Center for Biotechnology Information (NCBI) GenBank under the accession no. JAKKXF000000000.1 and automatically annotated by the Prokaryotic Genome Annotation Pipeline (PGAP) from NCBI. To assess the bile salt hydrolase activity of B. vulgatus Bv46 qualitatively, an agar plate assay was employed according to the method of Sui et al. (80) with slight modifications. Briefly, a fresh B. vulgatus Bv46 bacterial suspension of 20 μL with 10^9^ CFU mL^−1^ was spotted on BHI agar plates that contained additional taurodeoxycholic acid sodium salt (0.5% [wt/vol]) and calcium chloride (0.037%) and then incubated at 37°C for 5 days under anaerobic conditions. Lactobacillus gasseri Y20 (CGMCC no. 21252) and Salmonella enterica serovar Typhimurium 1344 (CGMCC no. 1.1174) served as positive- and negative-control strains (26), respectively.

### Animals and experimental regime.

Specific-pathogen-free (SPF) Sprague-Dawley (SD) rats (female; 200 ± 15 g) were purchased from Vital River Lab Animal Technology Co., Ltd. (Beijing, China). Animals were housed at two individuals per cage under a 12-h light-dark cycle condition controlled at a temperature of 23 ± 2°C and 55% ± 5% relative humidity, with free access to food and water. At the end of the acclimatization period, the rats were randomly separated into three groups (n = 8) with the following assigned diets: (i) the normal control (NC) group, a normal-chow diet with 2 mL (every other day) phosphate-buffered saline (PBS; 0.01 M, pH 7.4); (ii) the high-fat (HF) group, a high-fat diet (81) with 2 mL (every other day) PBS; and (iii) the B. vulgatus (BV) group, a high-fat diet plus intragastric gavage of B. vulgatus Bv46 suspended in PBS (1.0 × 10^9^ CFU mL^−1^, 2 mL every other day).

During the 6-week feeding period, body weight and food consumption were monitored weekly. At the end, fresh fecal samples were collected and frozen rapidly in liquid nitrogen. Thereafter, all rats underwent 12-h fasting and deep anesthetization for the following operations: whole blood from the abdominal aorta was drawn and centrifuged at 1,500 × *g* for 15 min at 4°C, and the obtained serum was snap-frozen for 15 min in liquid nitrogen. Simultaneously, the liver tissue was excised, weighed, and soaked in 4% paraformaldehyde or RNAlater solution. All the samples mentioned above were stored at −80°C before subsequent analysis. The animal experiment facility and protocols were approved by the Ethics Review Committee of the National Institute for Communicable Disease Control and Prevention at the Chinese Center for Disease Control and Prevention (approved no. 2021-019).

### Biochemical and histological analysis.

TC, TG, HDL-C, LDL-C, and liver cholesterol were measured using commercial diagnostic kits. Serum levels of cytokines, including IL-1β, IL-6, TNF-α, MCP-1, and ET, were evaluated with enzyme-linked immunosorbent assay kits. For histopathological analysis, liver tissue samples fixed in 4% paraformaldehyde were trimmed, dehydrated, and embedded in paraffin wax, followed by sectioning using a cryotome, and the 5-μm-thick slices were stained with hematoxylin and eosin (H&E) for subsequent pathological changes captured via 10× and 40× objective lenses (Pannoramic Desk/Midi/250/1000).

### 16S rRNA amplicon sequencing.

DNA of rat fecal samples was extracted using a MagPure stool DNA KF kit B (Magen, China) and quantified by a Qubit fluorometer (Invitrogen, USA). The hypervariable regions V3-V4 of bacterial 16S rRNA were chosen for amplicon sequencing using primers 341F (5′-ACTCCTACGGGAGGCAGCAG-3′) and 806R (5′-GGACTACHVGGGTWTCTAAT-3′). The thermal cycling was 3 min at 94°C followed by 30 cycles of 94°C for 30 s, 56°C for 45 s, and 72°C for 45 s and a final extension at 72°C for 10 min. The amplicon was purified with AMPure XP beads and qualified by the Agilent 2100 bioanalyzer (Agilent, USA), and the resulting libraries were subjected to sequencing on the Illumina HiSeq 2500 platform.

The bioinformatics analysis of the 16S rRNA gene profile followed the tutorial of the pipeline EasyAmplicon (v 1.14) (82). Briefly, paired-end sequencing reads were merged and filtered by VSEARCH (v 2.15) with the maximum expected error rate of 0.01. The filtered sequences were dereplicated and denoised into unique sequences (zero-distance operational taxonomic units [zOTUs]) with UNOISE implemented in USEARCH (v. 10). To remove chimeric sequences, a reference-based alignment of zOTUs against the SILVA database (v. 123) was performed. The characteristic sequences were taxonomically classified using the sintax algorithm based on the SILVA database. The R package MicrobiotaProcess was used to normalize the read counts across samples and calculate the α- and β-diversity. For zOTUs with a median relative abundance of >0.1%, taxonomic differences at genus level were evaluated. To estimate the presence and abundance of B. vulgatus in feces of rats after ingestion, quantitative real-time PCR analysis was performed as described previously (83, 84). In addition, PICRUSt2 (v. 2.5.0) was used to predict the microbial functional abundance based on the KEGG database. To identify discriminant pathways, STAMP (v. 2.1.3) was employed with two-sided Welch’s *t* tests, and the Benjamini-Hochberg method was used to control the false-discovery rate (FDR); the *q* values were set at 0.05.

### Nontargeted metabolite profiling.

An aliquot (100 μL) of plasma sample was individually mixed with prechilled 80% methanol and 0.1% formic acid and homogenized by vigorous vortexing, after which the mixture was bathed on ice for 5 min and centrifuged at 15,000 × *g*, 4°C, for 20 min. Subsequently, the supernatant was diluted to an ultimate density of 53% methanol with liquid chromatography-mass spectrometry-grade water and transferred to an Eppendorf tube for centrifugation at 15,000 × *g*, 4°C, for 20 min, and the resulting supernatant was analyzed with a Vanquish ultrahigh-performance liquid chromatography (UHPLC) system coupled with an Orbitrap Q Exactive TMHF-X mass spectrometer (Thermo Fisher, Germany). Chromatographic separation was carried out using a Hypersil Gold column (100 by 2.1 mm, 1.9 μm) with a 17-min linear gradient at a flow rate of 0.2 mL/min. Formic acid (0.1%) in water and methanol were used as eluents A and B for the positive polarity mode; for the negative polarity mode, the eluents were 5 mM ammonium acetate, pH 9.0 (eluent A), and methanol (eluent B). The gradient profile was set as follows: 2% B, 1.5 min; 2% to 100% B, 12.0 min; 100% B, 14.0 min; 100% to 2% B, 14.1 min; 2% B, 17 min. Mass spectra were obtained using the following parameters: spray voltage, 3.2 kV; capillary temperature, 320°C; sheath gas flow rate, 40 arb; and auxiliary gas flow rate, 10 arb.

The original peak data were pretreated with Compound Discoverer (CD3.1; ThermoFisher) and matched to the mzCloud, mzVault, and MassList database for quantification. Further multivariate analyses were performed using MetaAnalyst 5.0, and variable adjustments were attempted based on sum normalization, log transformation, and pareto scaling; after that, PLS-DA and OPLS-DA were performed. Generally, metabolites with a variable importance plot (VIP) value of >1 and a *P* value of <0.05 were considered differential metabolites and searched against the KEGG database for pathway analysis.

### BA profiling.

Fecal samples (100 mg) were grounded and resuspended with liquid nitrogen, and 100 μL of the diluted sample was added to 500 μL acetonitrile-methanol (8:2) and centrifuged at 12,000 rpm for 20 min to precipitate the protein. The clear supernatant was then dried with a nitrogen blower, and the residue was redissolved in 100 μL water-acetonitrile (8:2) with formic acid (0.1%) and centrifuged. The BA pool in supernatant (2 μL) was measured with an ultrahigh-performance liquid chromatography coupled to tandem mass spectrometry (UHPLC-MS/MS) system (AB Sciex Corp., USA). The separation was operated at a flow rate of 0.5 mL/min on an Agela Venusil MP C_18_ column (2.1 by 100 mm, 2.5 μm) with a stable temperature of 50°C. Analytes were eluted from the column by a linear gradient of 0.1% formic acid in water (solvent A) and acetonitrile (solvent B), and accurate mass detection proceeded in negative multiple-reaction mode (MRM) with the following parameters: ion spray voltage, −4,500 V; curtain gas pressure, 30 lb/in^2^; ion source temperature, 550°C; ion source gas pressure of 1 and 2, 65 lb/in^2^. BA calibrators of various concentrations were prepared by mixing stock solutions of individual bile acids and used to identify metabolites detected by LC-MS.

### SCFA quantification.

Thirty-milligram fecal samples were suspended in 900 μL 0.5% phosphoric acid solution, swirled for 2 min, and centrifuged at 14,000 × *g* for 10 min at 4°C. The supernatant (800 μL) was thoroughly mixed with an equal volume of ethyl acetate and centrifuged at 14,000 × *g* for 20 min at 4°C. The organic phase (600 μL) and 4-methylvaleric acid (500 μmol/L), which served as an external standard, were injected into an Agilent 7890A gas chromatograph coupled with an Agilent 5975C mass spectrometric detector (Agilent Technologies, USA) (85). The SCFA quantification was done in accordance with the standard curve.

### Gene expression analysis.

Total hepatic RNA from six individuals (three samples per group) was isolated using TRIzol reagent (Life Technologies, USA). The quality and quantity were assessed by a NanoDrop 2000 spectrophotometer and an Agilent 2100 bioanalyzer (Thermo Fisher Scientific, USA). RNA-Seq was conducted on the BGIseq-500 platform, Beijing Genomics Institute (Shenzhen, China). Reads passing filtration were quantified by the Salmon (v 1.8.0) tool, and identification of DEGs was performed by the DESeq2 package with a threshold of *P* < 0.01 and an absolute value of log_2_ fold change of >1.0. To annotate the pathways related to DEGs, the clusterProfiler package was utilized for GO enrichment analysis. Finally, gene set enrichment analysis (GSEA) (v 4.2.2) based on preranked genes was employed to exhaustively define the enriched KEGG gene set pathways (|normalized enrichment score (NES)| > 1, FDR < 0.05), where the KEGG gene set was gathered from the Molecular Signature Database (MSigDB) using the msigdbr package.

For quantification, 1 μg RNA was reverse transcribed and amplified using the PrimeScript reverse transcription (RT) reagent kit (TaKaRa, China) following the manufacturer’s guidelines. mRNA expression levels were determined by quantitative real-time PCR on a Rotor-Gene Q (Qiagen, Germany). The reaction mixture (20 μL) consisted of 10 μL of TB Green Premix *Ex Taq* II (TaKaRa, China), 0.8 μL of each primer (10 μmol/L), 1 μL of cDNA, and nuclease-free water. The amplification program was as follows: predenaturation at 95°C for 30 s, followed by 40 cycles at 95°C for 5 s and 60°C for 30 s. The gene expression levels were normalized to the housekeeping gene glyceraldehyde-3-phosphate dehydrogenase (GAPDH) and expressed as levels relative to the control. All of the genes and primer sequences are listed in Table S1 in the supplemental material.

### Statistical analysis.

Data were reported as mean ± standard error. Statistical evaluations and graphic plotting were performed using GraphPad Prism 9.0 and R 4.0 software. After distribution analysis with the Kolmogorov-Smirnov test, the experimental data were compared using one-way ANOVA with Tukey’s multiple-comparison test or the Kruskal-Wallis test with Dunn’s test. Differences were acknowledged statistically significant at a *P* value of <0.05, and a *P* value of >0.05 and <0.1 was considered a trend toward significance.

### Data availability.

The 16S rRNA amplicon sequencing data used in this study are publicly available at the NCBI Sequence Read Archive (SRA) database (accession number PRJNA846135). RNA-Seq data were uploaded to the NCBI-SRA (accession number PRJNA846278).
