# Supplementary material for: Bacteroides vulgatus Ameliorates Lipid Metabolic Disorders and Modulates Gut Microbial Composition in Hyperlipidemic Rats
Source: Microbiol Spectr. 2023 Jan 10;11(1):e02517-22. doi: 10.1128/spectrum.02517-22 (PMC9927244; doi:10.1128/spectrum.02517-22)
Supplement: Supplemental file 1 — Fig. S1 to S4. Download spectrum.02517-22-s0001.pdf, PDF file, 0.7 MB [file spectrum.02517-22-s0001.pdf]

***Bacteroides vulgatus* ameliorates lipid metabolic disorders and modulates gut microbial composition in hyperlipidemic rats**

Mingchao Xu[1,2†], Ruiting Lan[3†], Lei Qiao[2], Xiaoying Lin[1,2], Dalong Hu[3], Suping Zhang[1,2], Jing Yang[2,4], Juan Zhou[2], Zhihong Ren[2], Xianping Li[2], Guoxing Liu[2], Liyun Liu[2,4*] and Jianguo Xu[1,2,4,5*]

[1]Department of Epidemiology, Center for Global Health, School of Public Health, Nanjing Medical University, Nanjing 211166, Jiangsu Province, China.

[2]State Key Laboratory of Infectious Disease Prevention and Control, National Institute for Communicable Disease Control and Prevention, Chinese Center for Disease Control and Prevention, Beijing 102206, China.

[3]School of Biotechnology and Biomolecular Sciences, University of New South Wales, Sydney, New South Wales, Australia.

[4]Research Units of Discovery of Unknown Bacteria and Function, Chinese Academy of Medical Sciences, Beijing 102206, China.

[5] Institute of Public Health, Nankai University, Tianjin 300071, China

[†]These authors contributed equally to this work.

***Corresponding author:** Liyun Liu (liuliyun@icdc.cn) and Jianguo Xu (xujianguo@icdc.cn), State Key Laboratory of Infectious Disease Prevention and Control, National Institute for Communicable Disease Control and Prevention, China CDC, P.O. Box 5, Changping, Beijing, China.

SUPPLEMENTARY FIGURES AND LEGENDS

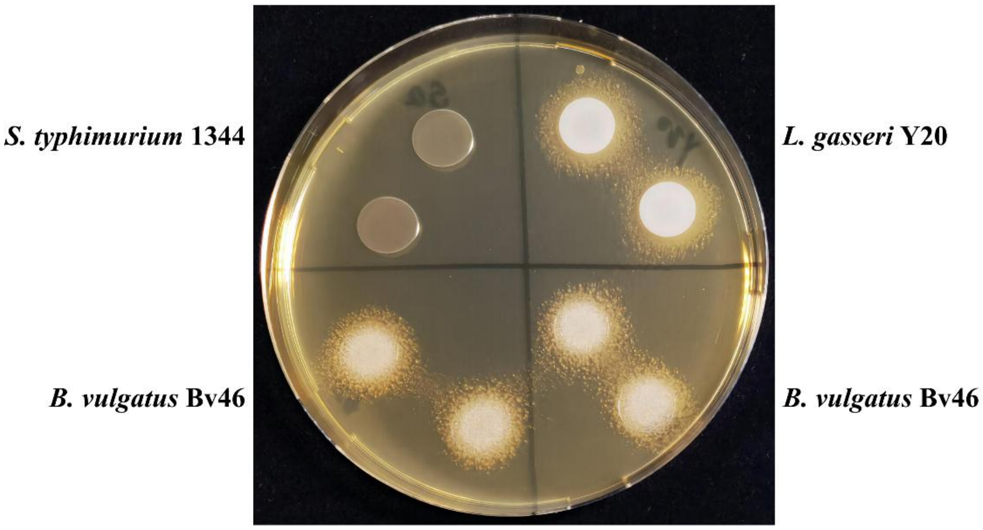

Fig. S1 Bile salt hydrolase activity of *B. vulgatus* Bv46 on BHI agar plate plus 0.5% taurodeoxycholic acid sodium salt.

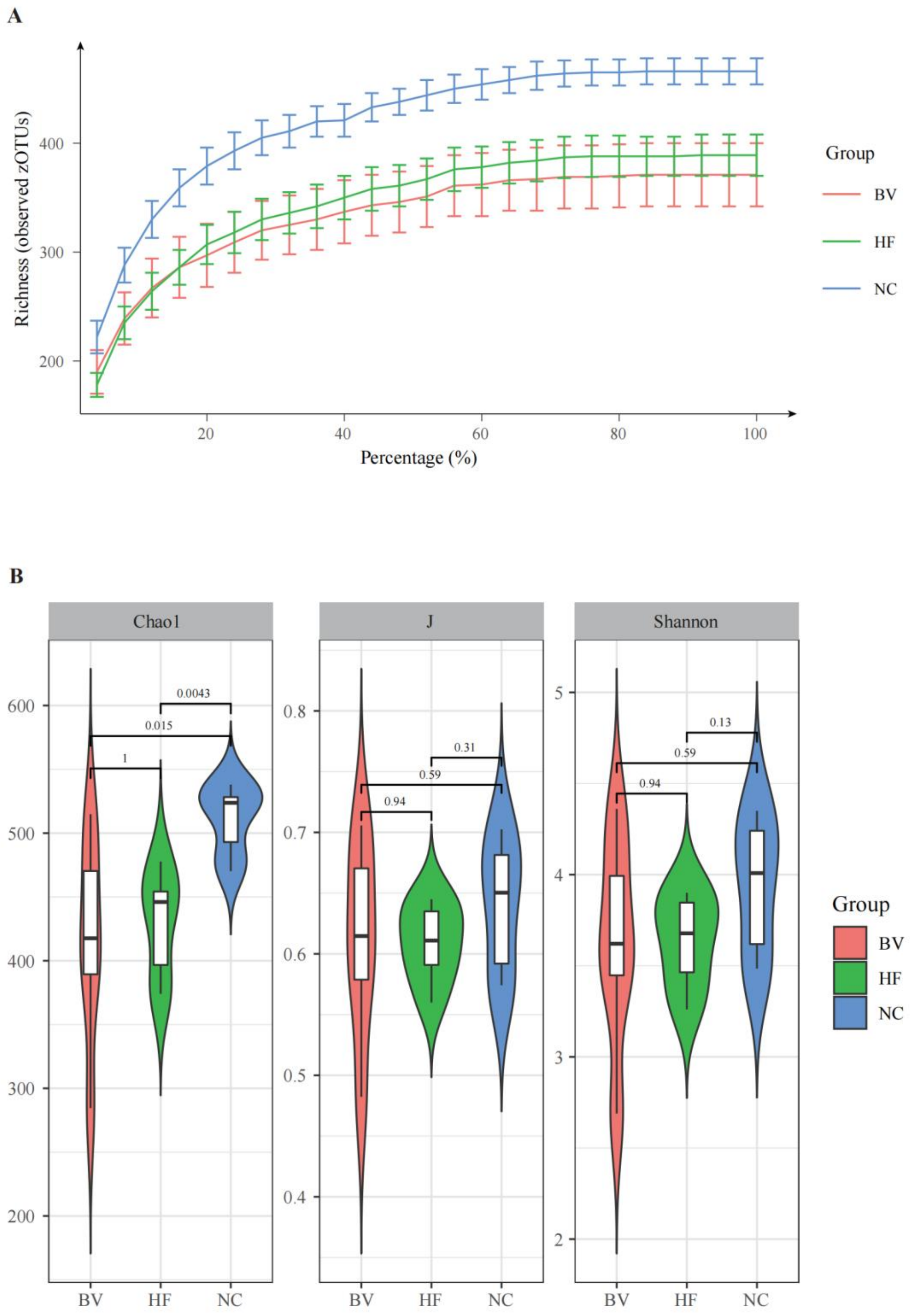
A
Richness (observed zOTUs)
200
300
400
20
40
60
80
100
Percentage (%)
Group
BV
HF
NC
B
Chao1
J
Shannon
600
500
400
300
200
0.0043
0.015
1
0.8
0.7
0.6
0.5
0.4
0.31
0.59
0.94
5
4
3
2
0.13
0.59
0.94
BV
HF
NC
Group
BV
HF
NC

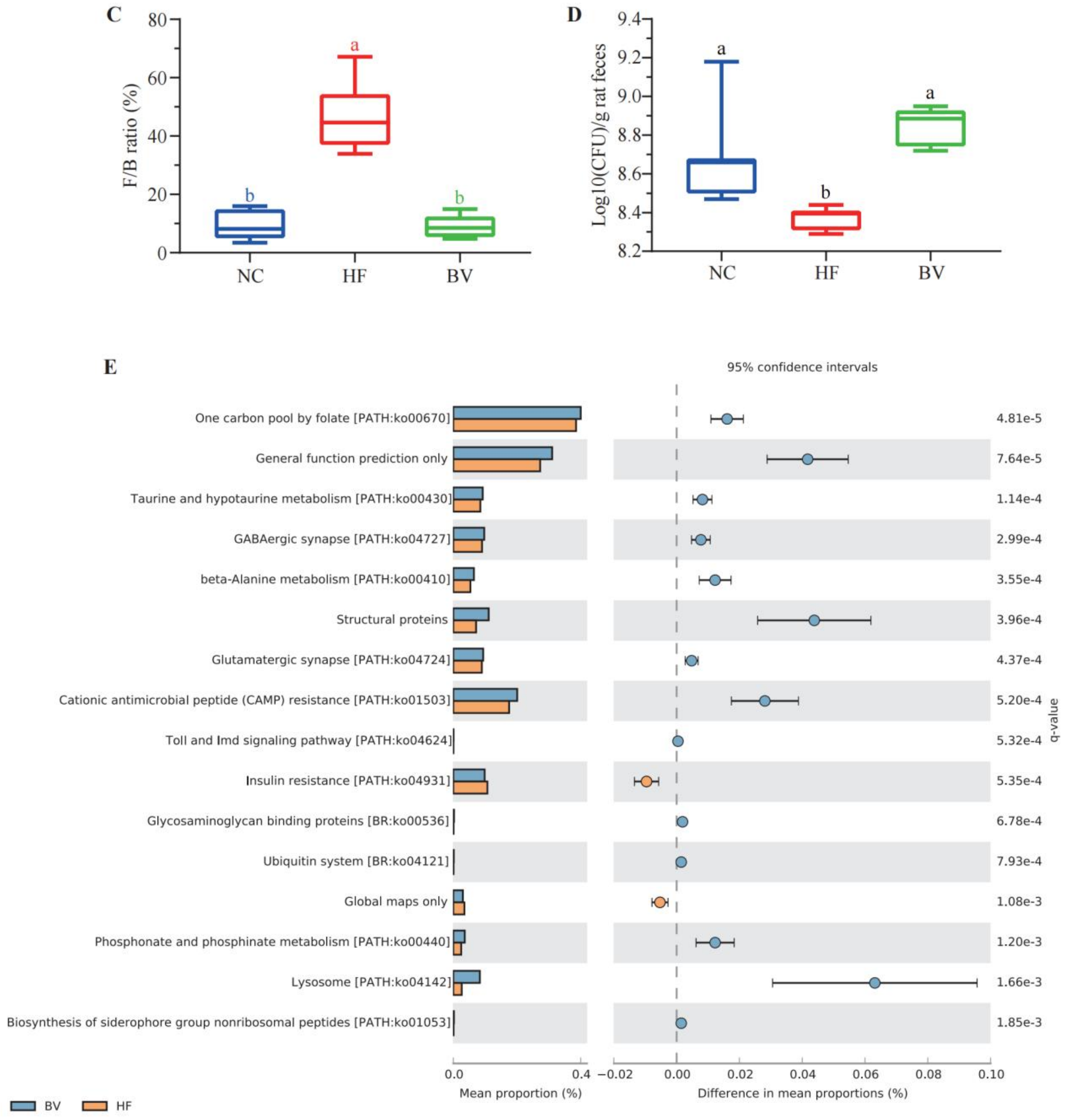

Fig. S2 Impact of different diets on the intestinal microbiota. (A) The rarefaction curve based on observed zOTUs in each group. (B) Measures of α-diversity indices: Chao1, J (Pielou evenness index) and Shannon. (C) *Firmicutes*/*Bacteroidetes* (F/B) ratio. (D) Quantitative biomasses of *B. vulgatus* in feces determined by species specific qPCR method. Statistical comparison was implemented by first testing normality with Kolmogorov-Smirnov test and then ANOVA with Tukey's multiple-comparison test or Kruskal-Wallis test with Dunn's post hoc test. Values are expressed as quartiles in each group, different letters (superscripts a-c) are considered as significantly different at $p < 0.05$. (E) Comparison of the functional predictions of the fecal microbiome between the BV and HF groups. The differences between groups were compared using Welsh's t-test, and the Storey's *q*-value was employed to control the false-discovery rate due

to multiple testing.

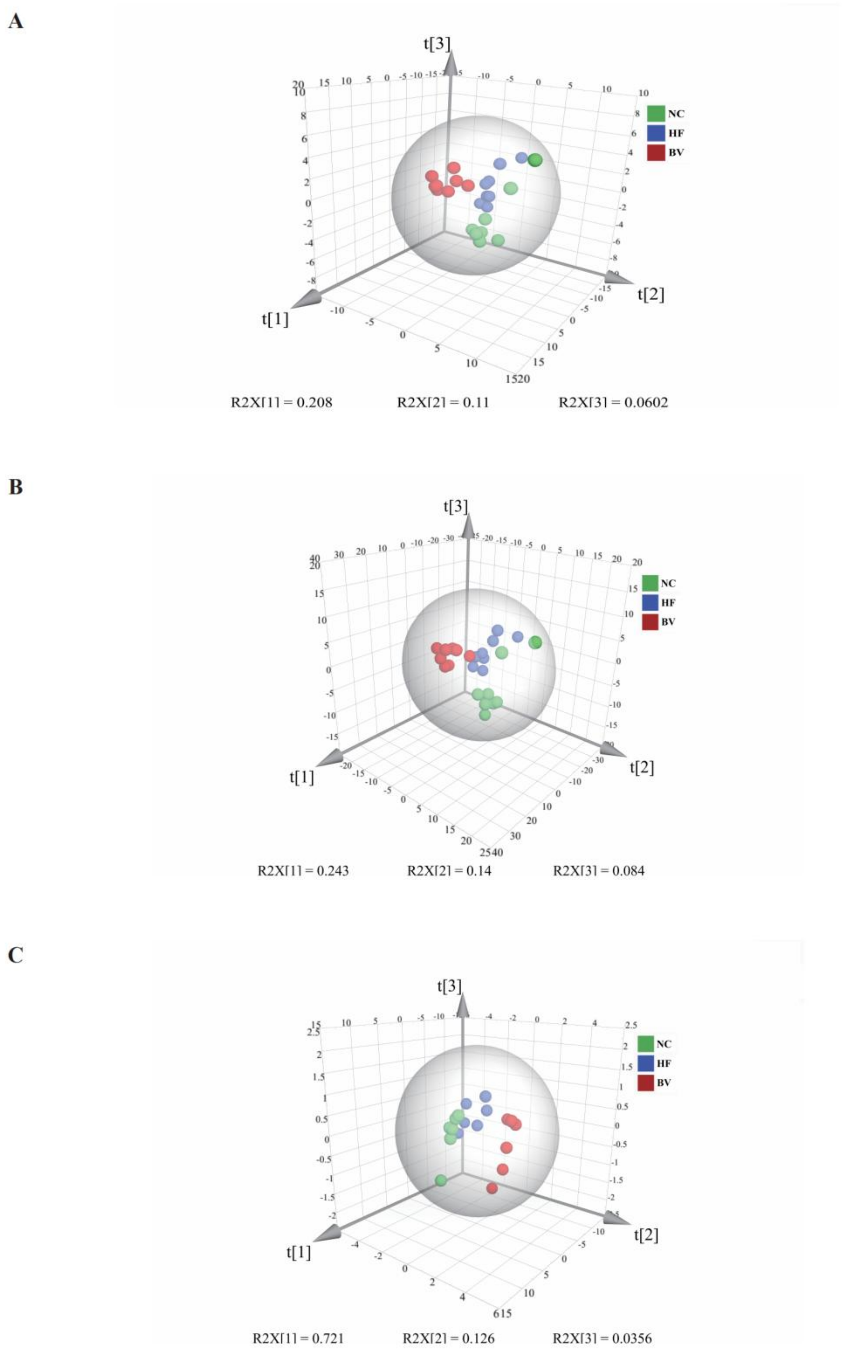

Fig. S3 3D partial least squares discriminant analysis (PLS-DA) scatter plots of the serum metabolic profiles classifying the three groups, (A) ESI+, (B) ESI-. (C) PLS-DA plot of bile acids profile in rats' fecal samples.

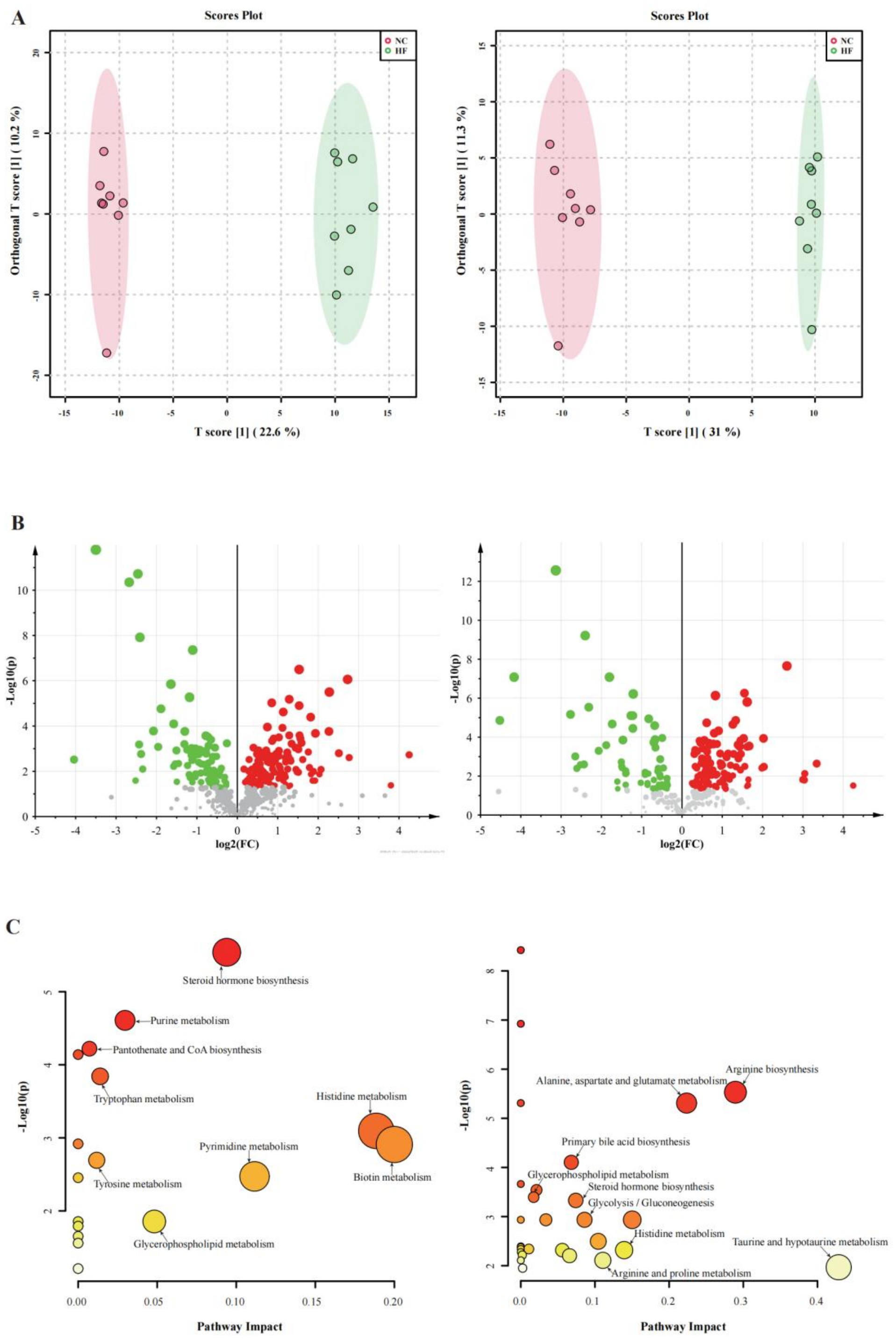

Fig. S4 Effect of the high-fat diet on metabolic profiles of serum in Sprague Dawley rats. ESI+: left column, ESI-: right column. (A) Orthogonal partial least squares discriminant analysis (OPLS-DA) plots of the NC and HF groups. (B) The volcano plots of the distribution of differential metabolites (ESI+: 216, ESI-: 148) in the serum. (C) 44 enriched KEGG pathways (*q*

< 0.05) plots of up- and down-regulated metabolites in the NC/HF group.
